# Cilia distribution and polarity in the epithelial lining of the mouse middle ear cavity

**DOI:** 10.1038/srep45870

**Published:** 2017-03-30

**Authors:** Wenwei Luo, Hong Yi, Jeannette Taylor, Jian-dong Li, Fanglu Chi, N. Wendell Todd, Xi Lin, Dongdong Ren, Ping Chen

**Affiliations:** 1 Department of Cell Biology Emory University, Atlanta, USA; 2 Eye, Ear, Nose, and Throat Hospital, Fudan University, Shanghai, PR China; 3 Electronic Microscopy Laboratory, Emory University, Atlanta, USA; 4 Center for Inflammation, Immunity and Infection, Institution for Biomedical Sciences, Georgia State University, Atlanta, USA; 5 Department of Otolaryngology, Emory University, Atlanta, USA.

## Abstract

The middle ear conducts sound to the cochlea for hearing. Otitis media (OM) is the most common illness in childhood. Moreover, chronic OM with effusion (COME) is the leading cause of conductive hearing loss. Clinically, COME is highly associated with Primary Ciliary Dyskinesia, implicating significant contributions of cilia dysfunction to COME. The understanding of middle ear cilia properties that are critical to OM susceptibility, however, is limited. Here, we confirmed the presence of a ciliated region near the Eustachian tube orifice at the ventral region of the middle ear cavity, consisting mostly of a lumen layer of multi-ciliated and a layer of Keratin-5-positive basal cells. We also found that the motile cilia are polarized coordinately and display a planar cell polarity. Surprisingly, we also found a region of multi-ciliated cells that line the posterior dorsal pole of the middle ear cavity which was previously thought to contain only non-ciliated cells. Our study provided a more complete understanding of cilia distribution and revealed for the first time coordinated polarity of cilia in the epithelium of the mammalian middle ear, thus illustrating novel structural features that are likely critical for middle ear functions and related to OM susceptibility.

Otitis media (OM), or inflammation of the middle ear, is the most commonly cited reason for visits to pediatricians^1^. Chronic otitis media with effusion (COME) is the leading cause of conductive hearing loss^2^,^3^,^4^,^5^. Primary Ciliary Dyskinesia (PCD) is a rare autosomal recessive genetic condition, about one in 10,000–30,000 individuals, which affects the function of motile cilia, and manifests as persistent secretion retention and chronic infection in the middle ear, nose and facial sinuses^6^,^7^,^8^,^9^. About 50% of the pediatric PCD cases were first suspected in ENT clinics during visits for COME^6^,^7^,^9^,^10^,^11^,^12^. The significant association between COME and PCD implicates the predilection of cilia dysfunction to COME and conductive hearing loss.

Despite the potentially significant role that cilia may play in middle ear function and pathology, ciliation in the middle ear epithelium is not well understood^13^,^14^,^15^,^16^,^17^. A recent study used genetic tracing tools to illustrate the dual origin of the epithelium lining the mammalian middle ear cavity, and determined the developmental timeline of the maturation of the middle ear cavity in mice^17^,^18^,^19^. While the epithelial cells lining the dorsal region of the middle ear cavity are derived from neural crests, the epithelium covering the ventral region of the middle ear cavity is formed from the endoderm-originated 1^st^ pharyngeal pouch^17^,^18^,^19^. Examination of the expression of acetylated α-tubulin, which is enriched in cilia axonemes, and of scanning electron microscopy (SEM) provided general divisions of ciliated and non-ciliated regions in the middle ear epithelium^17^. These data together suggest that the epithelium derived from the endoderm which covers the ventral region of the middle ear cavity near the Eustachian tube orifice is ciliated, while the epithelium derived from the neural crests which lines the dorsal region of the middle ear cavity is not ciliated^17^.

The polarity or the orientation of motile cilia critically underlies normal cilia functions^20^,^21^,^22^,^23^,^24^,^25^. In the trachea, motile cilia adorn the surface of epithelial cells and are uniformly oriented, which drives a directional outward flow that is critical for mucociliary clearance^23^,^26^,^27^. In the brain ventricles, motile cilia are also uniformly oriented to drive a directional cerebrospinal fluid flow that provides directional cues for brain development and is required for normal spine curvature^23^,^28^,^29^,^30^,^31^. The uniform orientation of motile cilia in these tissues is a manifestation of planar cell polarity (PCP)^26^,^32^,^33^,^34^,^35^,^36^, which refers to the coordinated polarization of cells along the plane of the tissue. The polarity and the beating direction of each cilium are dictated by the position and orientation of the basal body^21^,^23^,^27^,^37^,^38^. The basal body is a mother centriole-derived nine triplet (9 × 3) microtubule structure that acts as the foundation to the motile cilia axoneme, which consists of nine doublet (9 × 2) microtubules along with a central microtubule pair (9 × 2 + 2)^21^,^37^. In addition to the microtubules, the basal body consists of an appendage structure, the basal foot, which marks the orientation of the cilium and is composed of specific proteins that are electron dense on transmission electron microscopy (TEM) micrographs^21^,^27^,^38^,^39^. The polarity information of the motile cilia in the middle ear epithelium is unknown.

Here, we report on the distribution of cilia and the polarity of cilia in the mature mouse middle ear epithelium. We confirmed that the epithelium near the Eustachian tube orifice, which is developed from the endoderm-originated 1^st^ pharyngeal pouch^17^, is ciliated. We found that these cilia are coordinately oriented toward the Eustachian tube orifice. Surprisingly, we also found a second population of ciliated cells in the epithelium lining the middle ear cavity. This second population of ciliated epithelial cells cover the dorsal region of the middle ear cavity within the epithelium derived from neural crests^17^. This population of ciliated cells have the same composition of Keratin 5-posive basal cells and Keratin 5-negative multi-ciliated cells normally observed in the multi-ciliated mucosa^40^,^41^,^42^,^43^, including the middle ear ciliated epithelium near the Eustachian tube orifice. The cells in the non-ciliated epithelial regions are Keratin 5-positive. They form a thin, singular layer of cells which line the middle ear cavity between the two regions of ciliated cells at the ventral and dorsal poles of the middle ear cavity. These results provide new insights into the potential functions of cilia in the middle ear, and point to the importance of not only cilia but also cilial polarity in both the normal functioning and pathogenesis of the middle ear.

## Results

### Dual populations of ciliated cells in the mouse middle ear epithelium

During development, the endoderm-originated 1^st^ pharyngeal pouch extends to give rise to the epithelial layer of the Eustachian tube and the epithelial lining of the ventral region of the middle ear cavity, or the tympanic cavity, in mice^17^. The epithelial lining of the dorsal region of the middle ear cavity is derived from neural crest cells that fuse with the ruptured extending Eustachian tube during development^17^. By postnatal day 17 (P17) in mice, the formation of the middle ear cavity is complete. The fully formed middle ear cavity is covered with epithelial cells derived from both neural crests and the endoderm at its dorsal and ventral regions, respectively^17^.

**Figure 1:**
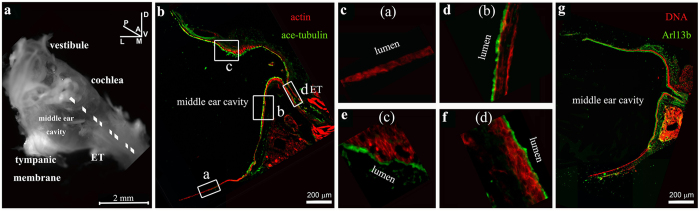
Cilia in the ventral region of the epithelial lining of the middle ear. (**a**) A dissected right temporal bone illustrates the relative position of the Eustachian tube (ET), the inner ear (cochlea and vestibule), the middle ear cavity, and the tympanic membrane. The cryosection plane is denoted by the dashed line. The inner ear is on the other side the medial wall of the middle ear cavity and is dorsal to the Eustachian tube, while the tympanic membrane is on the lateral side of the middle ear cavity. The vestibular structure is dorsal to the cochlea in the inner ear. The orientation designations are: L-lateral; M-medial; V-ventral; D-dorsal; A-anterior; P-posterior. Scale: 2 mm. (**b**–**g**) Temporal bone cryosections staining with an antibody against acetylated α-tubulin (green) and phalloidin (red) for actin (**b**–**f**), or an antibody against Arl13b (green) and Hoechst for DNA (red) (**g**). Panels **c**-**f** represent the larger views of the boxed regions (**a–d**) in **b**, in the epithelium of the middle ear at the areas of the tympanic membrane (**a**), ventral regions near the ET (**b**) and (**c**), or in the ET (**d**). The ciliated areas consist of a lumen staining for acetylated α-tubulin that is enriched in the cilia axoneme. The ciliation pattern in the middle ear revealed by acetylated α-tubulin staining (**b**) was validated by Arl13B staining (**g**), which marks the axoneme. Scale (**b**,**g**): 200 μm.

**Figure 2:**
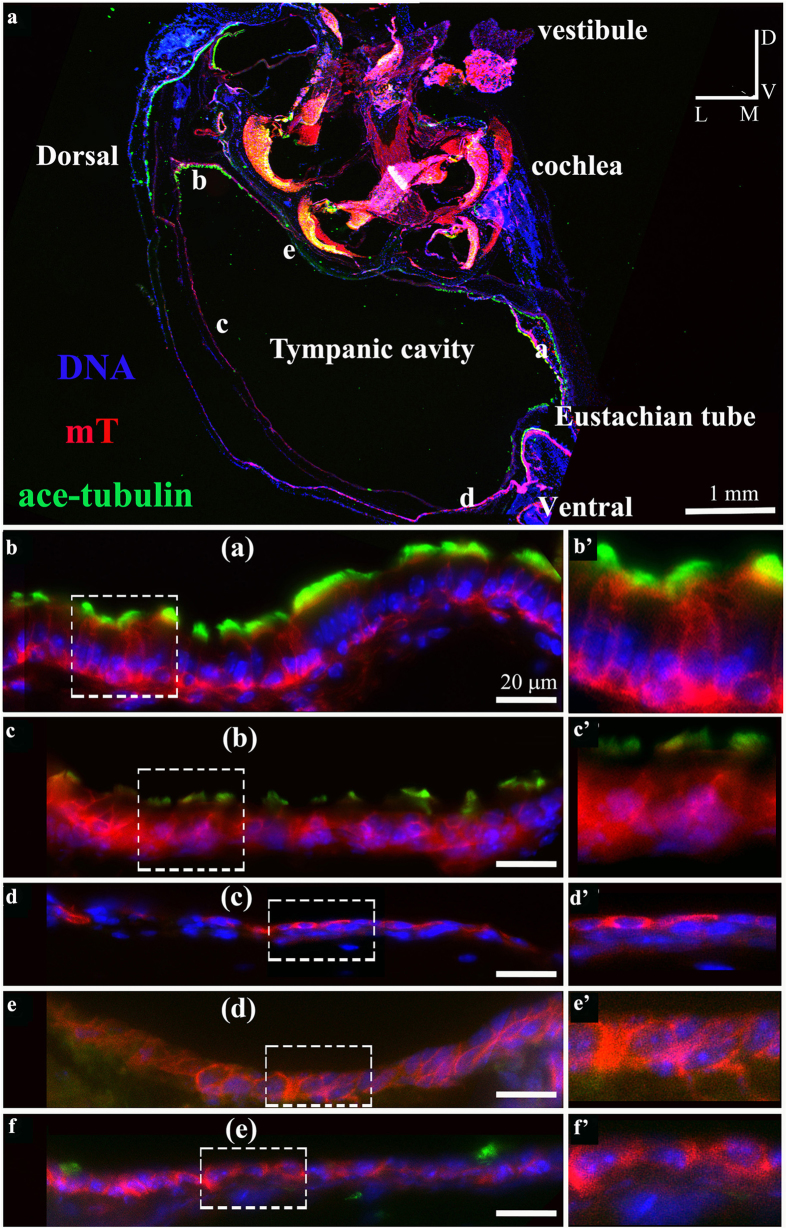
Dual ciliated regions in the epithelium that lines the middle ear cavity. (**a**–**f**) A temporal bone from an adult mouse expressing the red fluorescent protein membrane Tomato protein (mT) was cryo-sectioned and staining for cilia using an antibody against acetylated α-tubulin (green) and DNA (Hoechst, blue). Ciliation was detected in both the ventral region and the dorsal region of the epithelium that lines the middle ear cavity. Panels (**b)** to (**f)** correspond to regions (**a**–**e**) in (**a)**. (**b’**)–(**f’**) are larger views of the boxed regions in corresponding (**b)**–(**f)**. The presence of cilia in the dorsal region was verified by examination of serial sections (Fig. S1). Scale: 1 mm (**a**) and 20 μm (**b**–**f**). The orientation designations in (**a**) are: L-lateral; M-medial; V-ventral; D-dorsal.

**Figure 3:**
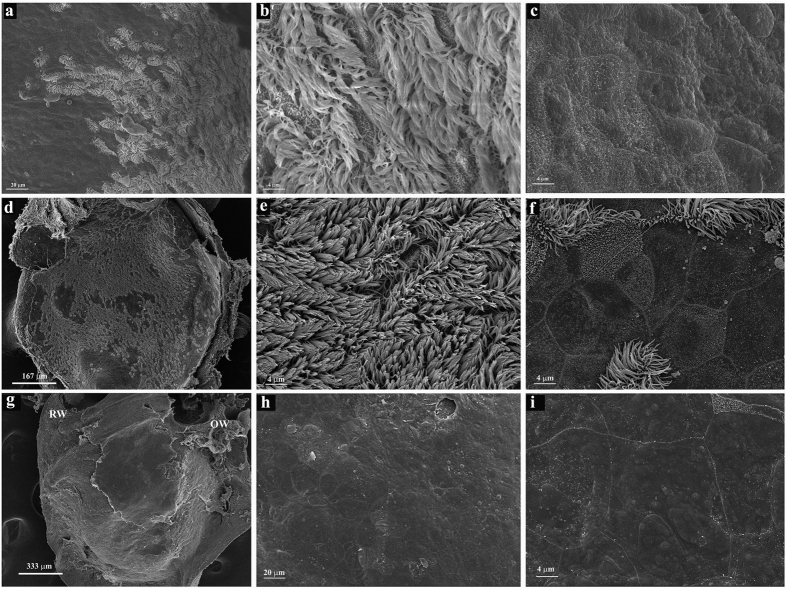
Scanning electron microscopy of the middle ear epithelium. (**a**–**c**) Scanning electron microscopy (SEM) of the middle ear from an adult mouse (4 months old) near the orifice of the Eustachian tube, which is located outside the right bottom corner of the image (**a**). The region consists of mostly multi-ciliated cells (**b**), and is bordered by non-ciliated cells on the lateral wall of the tympanic or middle ear cavity (**c**). Scale bars: 20 μm (**a**), 4 μm (**b**,**c**). (**d**–**f**) SEM of the posterior dorsal roof regions of the middle ear cavity above the round window. Extensive ciliation was observed in the region (**d**–**f**). Scale bars: 167 μm (**d**), 4 μm (**e**), 4 μm (**f**). (**g**–**i**) The region lining the promontory of the cochlea, or the bulging of the medial wall of the tympanic cavity produced by the cochlea, which is medial and ventral to the dorsal roof of the middle ear cavity, is not ciliated and shares similarity to the non-ciliated region on the lateral wall of the tympanic cavity. The round window (RW) is posterior to the oval window (OW). The roof region dorsal to the round window is the region shown in (**d**–**f**). Scale bars: 333 μm (**g**), 20 μm (**h**), 4 μm (**i**).

We examined ciliation in mature mouse middle ears (Figs 1 and 3, S1). We isolated temporal bones from P30-P120 mice, decalcified the samples, prepared oblique transverse cryosections of the temporal bones (Fig. 1a), and stained the sections with an antibody against acetylated α-tubulin that was enriched in cilia axoneme^44^ (Figs 1b–f and 2, S1) and an antibody against Arl13b (Fig. 1g) that was localized to the cilia axoneme^45^. As expected^17^, the epithelium lining the ventral region of the middle ear cavity near the Eustachian tube orifice and the epithelium of the Eustachian tube were positive for acetylated α-tubulin and ciliated (Fig. 1b,d,e), while areas away from the Eustachian tube orifice (Figs 1b,c and 2, S1) were not ciliated. Scanning electron microscopy (SEM) similarly revealed that the epithelium lining of the ventral region of the middle ear cavity near the Eustachian tube was ciliated (Fig. 3a–c).

The epithelial cells that line the ventral region of the middle ear cavity near the Eustachian tube derive from the endoderm during development^17^. Our data on ciliation patterns in this region is consistent with that found in previous reports^14^,^16^, which point to the idea that endoderm-derived cells in the middle ear epithelium are ciliated^17^. It is thought that the epithelial cells lining the dorsal region of the middle ear cavity, which derive from neural crest cells, are not ciliated^17^. Surprisingly, however, we observed ciliation in this region (Fig. 2). We verified ciliation in the epithelium that lines the dorsal region of the middle ear cavity by examination of multiple serial sections (Fig. S1), and SEM (Fig. 3d–i). The epithelium lining the dorsal roof of the middle ear cavity above the round window is extensively ciliated (Fig. 3d–f), while the epithelial cells lining the medial wall of the middle ear cavity are not ciliated (Fig. 3g–i).

### Coordinated orientation of cilia in the mouse middle ear epithelium

In the respiratory epithelium of the trachea, motile cilia are aligned uniformly to generate a directional beating motion across the mucosal tissue for clearance^21^,^22^,^46^. Although the polarity of the motile cilia in the nasal epithelium has not been examined directly, their motility and kinetics^47^,^48^,^49^,^50^,^51^ suggest that cilia in the nasal respiratory epithelium are likely also aligned to generate synchronized beating waves for mucociliary clearance functions. Accordingly, it has been generally accepted that the cilia in the respiratory epithelia share common structural features - coordinated motility or polarity of the cilia in the respiratory epithelia – to allow for clearance functions. However, as with the nasal respiratory epithelium, the polarity of the cilia in the middle ear has not been directly examined.

**Figure 4:**
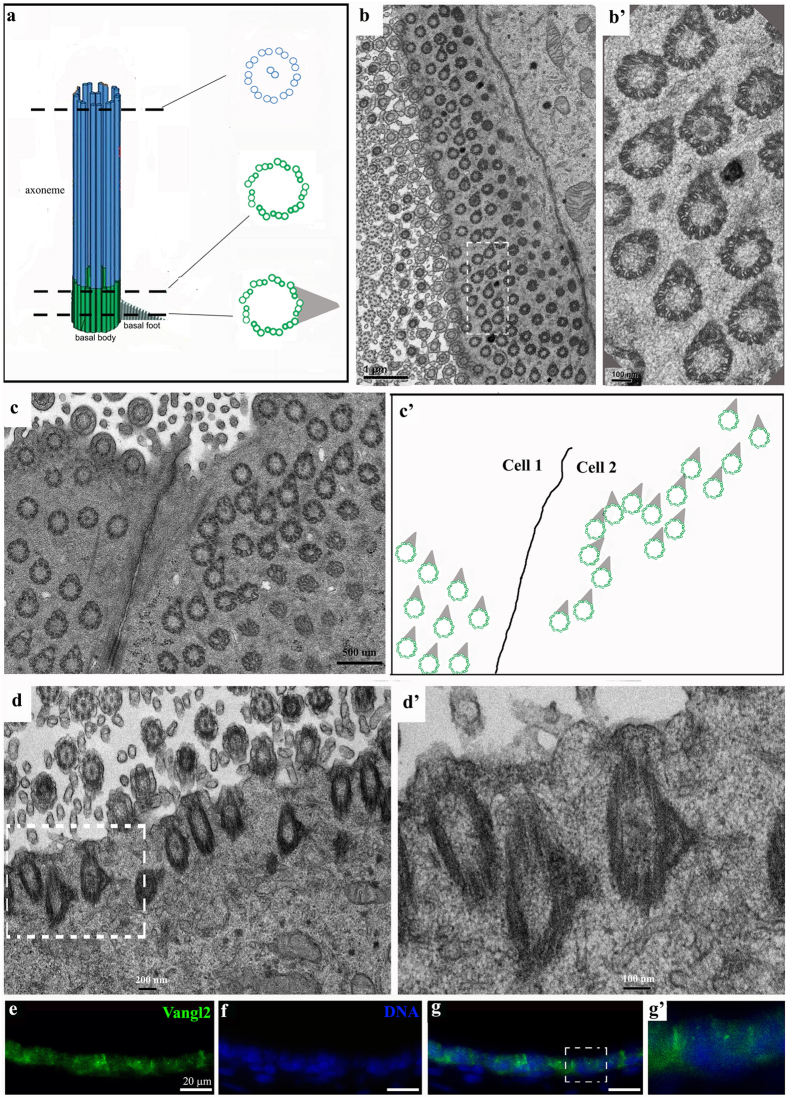
Planar cell polarity of the motile cilia in the middle ear. (**a**) Diagram of microtubules and the basal body of a motile cilium. The ciliary axoneme consists of nine tubulin doublets and a central pair of tubulin doublets that is the hallmark of a motile cilium. The basal body is made up of nine tubulin triplets. At the level of the basal foot, accessory proteins compose a triangular electron-dense structure on transmission electron microscopy (TEM) graphs, which marks the orientation of the cilium. (**b**–**d**) Horizontal (**b**,**b’,c,c’**) and vertical (**d**,**d’**) TEM micrographs of the middle ear epithelium. TEM through the basal foot level revealed that cilia are oriented toward the same direction (**b–d**). **b’** and **d’** are a large view of the boxed regions in (**b** and **d)** respectively. (**c’**) marks the orientation of basal feet when their orientation was visible in **c**. Scale bars: 1 μm (**b**), 100 nm (**b’**), 500 nm (**c**), 200 nm (**d**) and 100 nm (**d’**). (**e**–**g**) A cryosection of the middle ear staining with an antibody against Vangl2 (green) and DNA (Hoechst, blue). (**g’**) is a larger view of the boxed region in (**g**). Vangl2 is enriched at the apical cortex. Scale: 20 μm.

The polarity of motile cilia could be visualized by TEM of the electron dense accessory proteins in the basal foot of the basal body^21^,^27^,^37^ (Fig. 4a). We processed mouse temporal bones for thin sections along the horizontal plane and the vertical plane of the epithelium, and examined the sections using TEM (Fig. 4). The core microtubule structure in the axoneme portion of the motile cilia consist of nine doublets as well as a central pair, or 9 × 2 + 2 microtubules, while the microtubule structure in the basal body consists of nine triplets without the central pair, or 9 × 3 + 0 microtubules^52^ (Fig. 4a). Moreover, at the basal foot level, the basal body appears as a ring of nine triplets with an electron-dense triangle as visualized by TEM (Fig. 4). As revealed by TEM through the horizontal plane of the epithelium, the motile cilia in the middle ear epithelium near the Eustachian tube are oriented toward the same direction (Fig. 4b,b’). Similarly, the motile cilia in the middle ear epithelium at the dorsal roof of the middle ear cavity are also oriented toward the same direction (Fig. 4c,c’). The uniform orientation of motile cilia was also observed in TEM of vertical sections (Fig. 4d,d’).

The coordinated orientation of cells or cilia is a manifestation of planar cell polarity (PCP), and involves a set of conserved genes known as core PCP genes^20^,^32^,^53^. In particular, Vangl2 has been shown to be involved in all known PCP processes^20^,^32^,^54^,^55^. For PCP regulation in epithelial tissues, Vangl2 and other membrane or membrane-associated essential PCP proteins form membrane complexes at the cell-cell junction to coordinate the polarity of neighboring cells^36^,^56^,^57^,^58^. Indeed, Vangl2 is expressed in the middle ear epithelium (Fig. 4e–g,g’), and Vangl2 protein was localized to the apical cell-cell contacts in the ciliated epithelial cells in the middle ear (Fig. 4g’).

### Keratin 5 labels the basal cells of the ciliated regions, Goblet cells, and the non-ciliated cells in the middle ear epithelium

**Figure 5:**
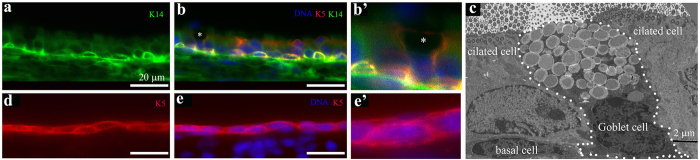
Keratin 5 positive cells in the epithelium lining of the middle ear cavity. (**a**–**c**) A cross section in the ciliated epithelium of the middle ear cavity, showing the presence of basal cells that are positive for Keratin 5 (K5, red)) and Keratin 14 (K14, green) (**a**,**b**,**b’**). DNA was stained by Hoechst (blue). (**b’**) is a larger view of a region in (**b**) marked by a * that appears to be a Goblet cell. Note that K5 also labeled the apparent Goblet cell while K14 did not. A TEM graph of the ciliated epithelium region revealed the high resolution cellular structure of the epithelium, consisting of the basal, ciliated, and Goblet cells (**c**). Note the distinct “goblet”-shaped morphology (**b**,**b’**,**c**) and the presence of numerous secretory vesicles in the Goblet cell (**c**). Note also that the Goblet cells do not contain cilia basal bodies or axoneme (**b**,**b’**,**c**), while thinner sections of microvilli of the Goblet cell are visible (**c**). Scale: 20 μm (**a**,**b**); 2 μm (**c**). (**d**–**e**) A cross section in the non-ciliated epithelium of the middle ear cavity, revealing a single layer of squamous epithelial cells that are positive for Keratin 5. (**e’**) is a larger view of a region in (**e**) Scale: 20 μm.

The cells lining the middle ear cavity appear to consist of two general epithelial types. The first is the ciliated epithelium, consisting mostly of a lumen layer of multi-ciliated cells and a basal layer of cells, while the second is the non-ciliated epithelium, consisting of a thin layer of squamous cells (Figs 1 and 3, S1). In the trachea and nasal mucosal epithelia, the basal layer of cells (aka “basal cells”), express the precursor-specific intermediate filament protein Keratin 5 and act as the precursor or the stem cells of the epithelia to give rise to and replenish the terminally differentiated multi-ciliated cells^40^,^41^,^42^,^43^. Similarly, Keratin 5 was observed in the basal cells of the ciliated epithelium in the middle ear (Fig. 5a,b and b’). It is also expressed in the Goblet cells within the ciliated region (Fig. 5a,b, and b’). Goblet cells were identified by their distinct stratified and goblet-shaped morphology in cryo-sections (Fig. 5a,b,b’) and by the presence of numerous secretory vesicles on TEM graphs (Fig. 5c). It is noted that previous studies have reported various characterizations of secretory cells in the middle ear epithelium, including Goblet cells and dark granulated cells^59^,^60^. Moreover, Keratin 5 is also expressed in the non-ciliated squamous epithelial cells in the middle ear (Fig. 5d,e,e’). Interestingly, while Keratin 14 is also expressed in the basal cells (Fig. 5a,b,b’) and the non-ciliated epithelial cells^17^, it is not in the Goblet cells (Fig. 5a,b,b’).

**Figure 6:**
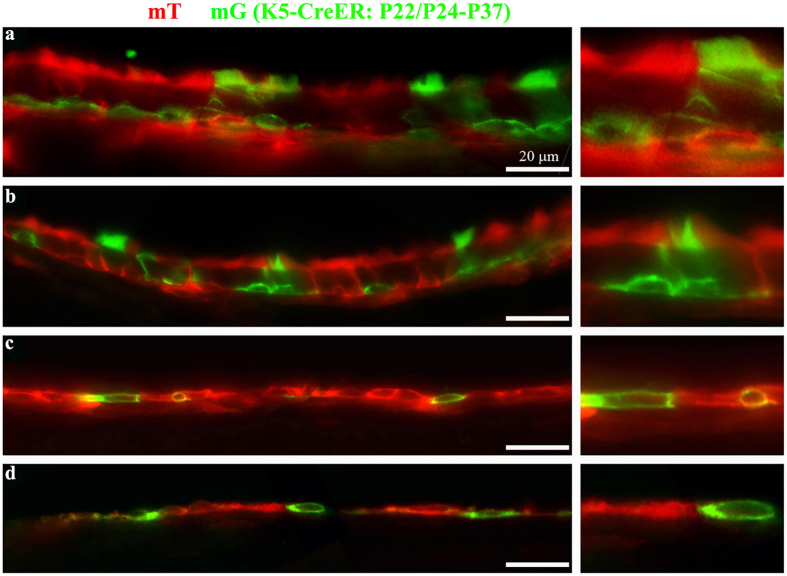
Keratin 5 positive cells replenish the ciliated and non-ciliated cells in the epithelium that lines the middle ear cavity. (**a**–**d**) Mice positive for Keratin 5 (K5) Cre-ER and mT/mG transgenes were injected with Tamoxifen at postnatal days 22 (P22) and P24. The temporal bones were collected at P37 and processed for visualization of the native mT and mG signals. Panels **a**-**d** represent regions in the epithelium from the ventral region near the orifice of the Eustachian tube (**a**), the dorsal ciliated region (**b**), the non-ciliated region against the inner ear (**c**), and the non-ciliated region against the tympanic membrane (**d**). Note that the basal cells are with a flat morphology at the basal layer of the epithelium, and that some ciliated cells are green, indicating their K5-positive origin. Scale: 20 μm.

We further used the Keratin 5-CreER line^61^ crossed with a reporter line, mT/mG^62^, to verify the presence of Keratin 5^+^ cells and trace Keratin 5^+^ cells in both the ciliated and non-ciliated epithelia (Fig. 6). We injected tamoxifen at P22 and P24, and collected the temporal bones at P37. The activation of CreER upon tamoxifen injections results in the conversion of membrane Tomato protein (mT) to membrane GFP (mG) in Keratin 5^+^ cells^61^,^62^. We observed GFP^+^ -basal cells and GFP^+^-ciliated cells in both ciliated regions of the epithelium lining the middle ear cavity (Fig. 6a,b). We also observed GFP^+^ cells in the non-ciliated regions (Fig. 6c,d). The data confirmed that Keratin 5^+^ basal cells give rise to ciliated cells in the two ciliated regions in the middle ear (Fig. 6a,b), and that Keratin 5^+^ squamous cells in the single cell layer of the non-ciliated epithelium of the middle ear replenish the epithelial cells in those regions (Fig. 6c,d).

## Discussion

Motile cilia can provide motility for the cells, or generate a directional flow of the media across the tissues to provide directional cues or carry out clearance functions^20^,^21^,^23^,^25^. In the respiratory epithelium of the trachea, motile cilia are uniformly orientated to generate an outward flow for mucociliary clearance^27^. Cilia dysfunctions have been implicated in a number of conditions, including neonatal respiratory distress, chronic nasal congestion, bronchiectasis and sinusitis, and chronic otitis media with effusion^6^,^7^,^8^,^10^,^11^,^12^,^63^,^64^,^65^,^66^. Despite the significant association and likely contributory role of cilia in OM^6^,^7^,^11^,^12^,^63^,^66^,^67^, the most common childhood disease, only a sparse number of studies examined ciliation in the epithelium that lines the middle ear cavity and thus have provided only a partial understanding of ciliation in the middle ear^13^,^14^,^16^,^17^,^68^,^69^,^70^. Moreover, the orientation of the cilia, which is the structural feature of cilia critical to their motility output and clearance function, has not even been studied. In this study, we characterize ciliation in the epithelium that lines the middle ear cavity, and examine the orientation of these motile cilia of the middle ear.

We confirmed the presence of ciliation in the epithelial region near the orifice of the Eustachian tube at the ventral side of the middle ear (Figs 1 and 3, S1). We showed that this epithelium is made of typical Keratin 5^+^ basal cells and the lumen layer of multi-ciliated cells (Figs 5 and 6), and that the Keratin 5^+^ basal cells give rise to the lumen layer of ciliated cells (Fig. 6). Furthermore, we showed for the first time that the motile cilia in the region are aligned toward the general direction of the opening of the Eustachian tube (Fig. 4), and the planar cell polarity (PCP) protein Vangl2 is expressed in the ciliated epithelium (Fig. 4).

Moreover, we also identified a new population of ciliated cells in the epithelium lining the middle ear cavity. We found that the epithelial region at the dorsal roof of the middle ear cavity above the oval window is also ciliated via the examination of multiple serial sections (Figs 1 and 2, S1, 5, 6). The ciliated epithelium in the dorsal region also consists of Keratin 5^+^ basal cells and a lumen layer of multi-ciliated cells, and the Keratin 5^+^ basal cells give rise to the lumen ciliated cells (Figs 5 and 6). It would be of interest to explore the capability and time course of replenishment of ciliated cells by Keratin 5^+^ basal cells during pathology of the middle ear. Cell lineage determination using neural crest-specific and endoderm-specific Cre mouse strains in combination with a reporter strain indicated that the epithelium in the dorsal region derive from the neural crests while the epithelium in the ventral region originates from the endoderm^17^. Our data suggested that the neural crest derived epithelium in the middle ear could also develop into a typical mucosal epithelium that consists of a basal cell layer and a ciliated cell layer. Alternatively, the dorsal roof region above the round window is a continuation of the endoderm-derived epithelial region, as the determination of the exact boundaries of the two-lineage-derived epithelia may not be available due to limitations of markers and lineage tracing tools.

The significance of dual regions or extended regions of ciliated cells in the middle ear cavity at two poles of the middle ear cavity is perhaps two-fold. Firstly, the normal functions of the middle ear cavity include balancing air pressure and conducting sound signals to the inner ear^71^,^72^. The presence of motile cilia at the two poles of the middle ear cavity may present mechanical advantages for the efficacy of air flow and middle ear ossicle conductance of mechanical sound signals. Secondly, the same mechanical advantage afforded by the presence of cilia at the two poles of the middle ear cavity may also be beneficial for fluid and pathogen clearance from the middle ear.

In addition to functions associated with cilia motility, cilia in the human airway epithelia could also function in chemosensory-based defense mechanisms^73^,^74^,^75^. It will be of great interest to determine whether cilia in the middle ear also express chemosensory receptors and whether the two populations of ciliated cells in the middle ear have differential expressions of chemosensory receptors and act differentially in chemosensory related mechanisms.

The demonstration of motile cilia PCP in the middle ear points to the possibility that defective PCP signaling may lead to respiratory diseases and chronic OM with effusion, which is commonly associated with PCD and other ciliopathy diseases^6^,^7^,^12^,^64^,^65^,^66^,^76^,^77^. Our study supports the justification to expand upon the capacity of current tests for PCD and other ciliopathy-related diseases, which are considered to significantly underestimate the cases, to include the ability to reveal defects in PCP genes and cilia polarity^10^,^65^,^77^,^78^. The inclusion of PCP and cilia polarity genes in the genetic and pathological-morphological tests will likely lend support to the idea of PCP genes as a novel class of susceptibility genes for COME and other respiratory diseases, caused by a reduction or ablation of mucociliary functions of the motile cilia.

## Methods

### Animals

Wild type mice, mice carrying a mT/mG transgene or the *Gt(ROSA)26Sor*^*tm4(ACTB-tdTomato,-EGFP)/Luo*^/J (Jackson Laboratory stock#: 007576; mT/mG)^62^, and mice carrying K5-Cre-ERT2 transgene or *Tg(KRT5-cre/ERT2)2lpc*/JeldJ (Jackson Laboratory stock#: 018394; K5-Cre-ER^T2^)^61^ were used. Animal care and use was in accordance with NIH guidelines and was approved by the Animal Care and Use Committee of Emory University.

### Dissection of temporal bones, decalcification, and cryo-sectioning

The temporal bones of mice were dissected, and fixed in 4% PFA. The fixed temporal bones were decalcified in 10% EDTA for 24 hours or longer at 4° C. The decalcified samples were cryo-protected in 30% sucrose, embedded in OCT, and sectioned at 12 μm in thickness along an oblique transverse plane as diagramed in Fig. 1.

### Immunohistochemistry on cryosections

Immunostaining and imaging were performed as previously described^36^,^79^. Briefly, the sections were rehydrated, blocked with 10% donkey serum at room temperature for 30 minutes or longer, incubated with primary antibodies in PBS containing 0.1% Triton X-100 (PBST) overnight at 4° C, washed in PBST for three times at room temperature, incubated with appropriate fluorescent secondary antibodies at room temperature for 1 hour or longer, and mounted in Fluro-G (SouthernBiotech) for imaging. An inverted Olympus IX71 microscope or Zeiss confocal microscope was used for imaging.

The primary antibodies used were mouse against acetylated α-tubulin (Sigma, Cat#: T7451, 1:400), rabbit against Arl13b (gift from Tamara Caspary, Emory University, USA, 1:500)^45^, rabbit against Keratin 5 (BioLegend, Cat#: 905501, 1:500), mouse against Keratin 14 (ThermoFisher, Clone LL004, Cat#: MS-115-P0, 1:1000), and sheep against Vangl2 (R&D systems, Cat#: AF4815, 1:200). For staining with antibodies against Keratin 5, Keratin 14, and Vangl2, the sections were pretreated with an antigen retrieve procedure by incubating the sections in 10 mM citric buffer (pH 6.0) in a steamer for 10 minutes. In addition, Hoechst (ThermoFisher, Cat#: 33342, 1:10000) staining of DNA was included in most staining procedures to outline the cell layers in epithelia.

The antibody specificity for each of the antibodies for the specific cellular compartment(s) was confirmed using the corresponding pre-immune serum and by staining with cochleae and respiratory epithelia in the nose and trachea, in which the specificity of these antibodies has been demonstrated in numerous published studied including the studies using antibodies against Vangl2^80^, acetylated α-tubulin and Arl13b^36^,^45^, and Keratin 5 and Keratin 14^42^,^81^,^82^.

### Keratin 5-positive cell tracing

Animals carrying *K5-CreERT;mT/mG* were injected with Tamoxifen at postnatal day 22 (P22) and P24. Animals were sacrificed at P37. Temporal bones were collected and processed for cryosection and imaging of mT and mG native signals of the sections.

### Scanning electron microscopy (SEM) and transmission electron microscopy (TEM)

Temporal bones were fixed with 2.5% glutaraldehyde in 0.1 M cacodylate buffer (pH 7.4). Samples were then washed in 0.1 M cacodylate buffer followed by the post fixation with 1% osmium tetroxide in 0.1 M cacodylate buffer for 1 hour. After washing with de-ionized water, samples were dehydrated through an ethanol series and eventually placed in 100% ethanol. Samples were then placed into labeled specimen capsules and transferred to a sample holder filled with fresh 100% dry ethanol. The sample holder was placed into a pre-chilled Polaron E3000 critical point drying (CPD) unit and sealed. The CPD unit was filled with liquid carbon dioxide, which gently flowed through the chamber until the ethanol had been replaced with liquid carbon dioxide. The carbon dioxide was brought to its critical point of 1073 psi and 31 °C and allowed to gently bleed away. The dry samples were secured to labeled SEM stubs and sputter coated with 15 nm gold palladium using a Denton Vacuum Desk II sputter coater. The samples were imaged using a Topcon DS150 field emission scanning electron microscope at 10 kV.

For TEM, temporal bones were fixed with 2.5% glutaraldehyde in 0.1 M cacodylate buffer (pH 7.4). Samples from older mice were decalcified in 10% EDTA for a few days after fixation. Samples were then washed in 0.1 M cacodylate buffer and post-fixed with 1% osmium tetroxide for 1 hour. After washing with de-ionized water, samples were dehydrated through an ethanol series to 100% ethanol, infiltrated with Eponate 12 resin (Ted Pella, Inc., Redding, CA), placed in labeled Beam capsule with resin, and then polymerized in a 60 °C oven. Ultrathin sections were cut at 70–80 nanometer thick on a Leica UltraCut Sultramicrotome (Leica Microsystems Inc., Buffalo Grove, IL). Grids with ultrathin sections were stained with 5% uranyl acetate and 2% lead citrate. Ultrathin sections were imaged on a JEOL JEM-1400 transmission electron microscope (JEOL Ltd., Tokyo, Japan) equipped with a Gatan US1000 CCD camera (Gatan, Pleasanton, CA).

## Additional Information

**How to cite this article**: Luo, W. *et al*. Cilia distribution and polarity in the epithelial lining of the mouse middle ear cavity. *Sci. Rep.* **7**, 45870; doi: 10.1038/srep45870 (2017).

**Publisher's note:** Springer Nature remains neutral with regard to jurisdictional claims in published maps and institutional affiliations.

## Supplementary Material

Supplementary Information and Figure S1
