# Supplementary material for: Cilia distribution and polarity in the epithelial lining of the mouse middle ear cavity
Source: Sci Rep. 2017 Mar 30;7:45870. doi: 10.1038/srep45870 (PMC5372464; doi:10.1038/srep45870)
Supplement: Supplementary Information and Figure S1 [file srep45870-s1.pdf]

Cilia distribution and polarity in the epithelial lining of the mouse middle ear cavity

Wenwei Luo[1,2], Hong Yi[3], Jeannette Taylor[3], Jian-dong Li[4], Fanglu Chi[2*], N. Wendell Todd[5], Xi Lin[5], Dongdong Ren[2*], Ping Chen[1*]

Supplementary Information

Supplementary Figure Legend

Figure S1. Dual ciliated regions in the epithelial lining of the middle ear cavity

Serial sections of the temporal bone were examined to confirm the presence of a ciliated region at the dorsal pole of the middle ear cavity. One of the serial sections of figure 2 was shown (**a**). The orientation designations are: L-lateral; M-medial; V-ventral; D-dorsal. mT: membrane tomato protein nature signal (red). Panels **(b)** and **(c)** correspond to regions (a, b) marked in (**a**). (**b’**) and (**c’**) are larger views of regions in (**b**) and (**c**), respectively. Scale: 1 mm (**a**); 20 μm (**b**, **c**).

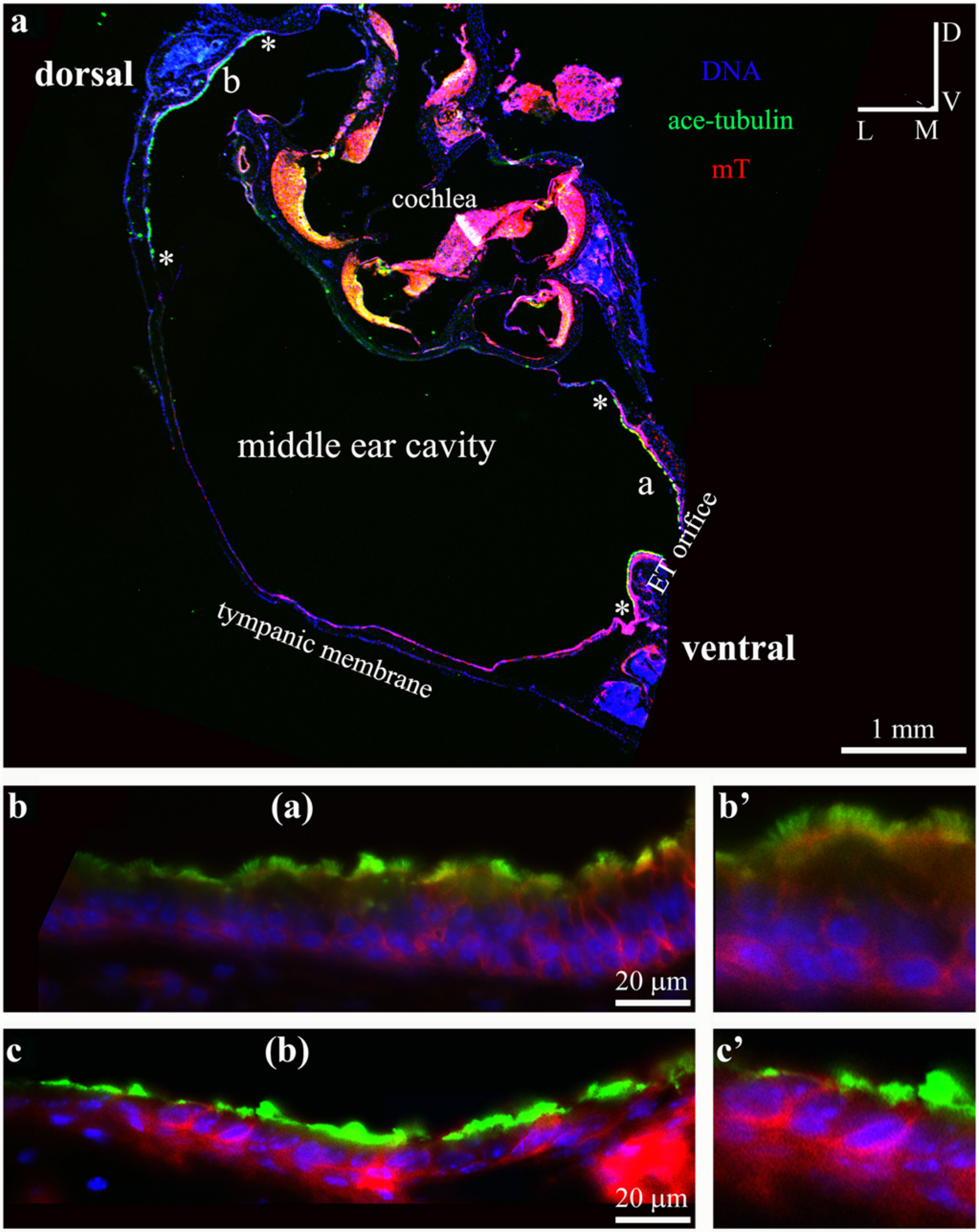

a
dorsal
b
*
DNA
ace-tubulin
mT
D
V
L
M
cochlea
*
*
middle ear cavity
a
ET orifice
*
tympanic membrane
ventral
1 mm
b
(a)
20 µm
b'
c
(b)
20 µm
c'
